# Online Prediction of Lead Seizures from iEEG Data

**DOI:** 10.3390/brainsci11121554

**Published:** 2021-11-24

**Authors:** Hsiang-Han Chen, Han-Tai Shiao, Vladimir Cherkassky

**Affiliations:** 1 Bioinformatics and Computational Biology, University of Minnesota, Minneapolis, MN 55455, USA; cherk001@umn.edu; 2 Department of Electrical and Computer Engineering, University of Minnesota, Minneapolis, MN 55455, USA; shiao003@umn.edu

**Keywords:** iEEG, non-stationarity, lead seizure, seizure prediction, support vector machines, unbalanced classification, group learning

## Abstract

We describe a novel system for online prediction of lead seizures from long-term intracranial electroencephalogram (iEEG) recordings for canines with naturally occurring epilepsy. This study adopts new specification of lead seizures, reflecting strong clustering of seizures in observed data. This clustering results in fewer lead seizures (~7 lead seizures per dog), and hence new challenges for online seizure prediction, that are addressed in the proposed system. In particular, the machine learning part of the system is implemented using the group learning method suitable for modeling sparse and noisy seizure data. In addition, several modifications for the proposed system are introduced to cope with the non-stationarity of a noisy iEEG signal. They include: (1) periodic retraining of the SVM classifier using most recent training data; (2) removing samples with noisy labels from training data; and (3) introducing a new adaptive post-processing technique for combining many predictions made for 20 s windows into a single prediction for a 4 h segment. Application of the proposed system requires only two lead seizures for training the initial model, and results in high prediction performance for all four dogs (with mean 0.84 sensitivity, 0.27 time-in-warning, and 0.78 false-positive rate per day). The proposed system achieves accurate prediction of lead seizures during long-term test periods, 3–16 lead seizures during a 169–364 day test period, whereas earlier studies did not differentiate between lead vs. non-lead seizures and used much shorter test periods (~few days long).

## 1. Introduction

There is a growing number of studies on data-analytic modeling for prediction and detection of epileptic seizures from intracranial electroencephalogram (iEEG) recordings. In recent years, seizure detection has become a mature technology. Several studies in seizure detection [1,2,3,4,5,6,7] demonstrate remarkable performance. Accurate seizure detection capability is critical for seizure prediction, because prediction relies on properly annotated (~labeled) training data. However, there are no comparable technologies that can achieve accurate *seizure prediction*, in particular online prediction. Furthermore, existing seizure prediction studies do not address prediction of *lead seizures* (i.e., the first seizure in a cluster). Such seizure clusters are evident from observation of all seizures detected over long-term period (~1 year) [8]. In this study, we focus on the development of an online prediction system for lead seizures.

Due to great variability of seizure patterns and modalities, the notion of ‘lead seizure’ is usually defined in a somewhat ad hoc manner, even though the existence of seizure clusters is well-known. In particular, seizure clustering is usually observed in naturally occurring seizures. Such seizures do not have known clinical causes (such as brain damage, stroke, etc.), and they often do not respond well to medication. This paper presents a system for prediction of lead seizures, using an available data set of long-term iEEG recordings of naturally occurring seizures. Specific clinical decisions or patients’ actions in response to warnings provided by a seizure prediction system are outside the scope of the paper. However, the effectiveness of all such decisions and actions obviously depends on the quality of predictions. In particular, the effectiveness of clinical actions in response to prediction of the first seizure in a cluster is more important (than for non-lead seizures).

All seizure prediction studies assume that there are changes in statistical characteristics of the iEEG signal preceding seizures [9,10,11,12,13,14]. Under this assumption, seizure prediction can be formalized via a binary classification setting [15,16,17,18,19], so that all iEEG segments are labeled as interictal or preictal. Preictal training segments correspond to iEEG recordings from a certain period (typically, ~10–360 min) before seizure onset; interictal segments are represented by iEEG recordings during a seizure-free period. Available iEEG data from past recordings is used to train a binary classifier. Then a trained classifier is used for predicting or forecasting future seizures (from iEEG input segments). There are two important considerations for online seizure prediction:

• First, most of the recent successful seizure prediction models are *subject-specific*, i.e., a good predictive model for particular patient should be trained using past iEEG data from that patient [18,20].
• Second, naturally occurring seizures are usually clustered in time. That is, multiple seizures occur within short time period (~cluster), and the time between successive clusters is much longer than duration of a cluster. It is clinically important to predict the first seizure in a cluster, also known as the lead seizure [21].

There is no common agreement on how to formally identify lead seizures in available data, because clusters are identified by visual analysis of seizure data currently performed by humans. Chen and Cherkassky [8] discovered certain stable patterns of seizure clusters in time, based on analysis of naturally occurring seizures in a publicly available canine data set. According to this analysis, the duration of seizure clusters is at most 3 days for all canines (patients) in this data set. That is, naturally occurring seizure clusters are separated by an at least 3-day seizure-free period. It leads to specification of lead seizures, as seizures preceded by (at least) 3 days seizure-free period. Using such a data-driven definition of lead seizures results in two additional challenges for predictive modeling. First, using smaller number of lead seizures reduces the amount of preictal data available for training. This makes modeling especially difficult for methods requiring a large amount of data (e.g., deep learning). Second, predicting the first seizure in a cluster is harder than predicting other seizures [8]. These new challenges have not been properly addressed in previous studies.

From a machine learning point of view, binary classification setting for predicting lead seizures should incorporate provisions for unbalanced data and non-stationarity of iEEG signal. The imbalance problem is due to small amount of preictal data (because seizures are rare events, and also due to subject-specific modeling). Unbalanced setting requires appropriate modifications during training and model selection. Earlier seizure prediction studies addressed this problem by using different misclassification costs [15,16,19,22].

Non-stationarity of the iEEG signal [18,23,24] makes seizure prediction especially challenging since all data-analytic modeling approaches assume statistical similarity between training and test data. This non-stationarity of the iEEG signal has not been properly addressed in earlier literature for online prediction.

The online seizure prediction system described incorporates the group learning method [16], for improved learning with very sparse high-dimensional data. This method can be incorporated into any classifier (SVM is used in this study). The group learning approach effectively reduces (input) dimensionality and increases the number of training samples. In the context of online seizure prediction, the goal is to make predictions for 4 h segments. However, each 4 h segment of iEEG signal is regarded as a group of many small-size windows (~20 s windows), so that:

• An SVM classifier is trained on windows data (yielding larger number of labeled training windows);
• The number of input features (for encoding 20 s window) is much smaller than the number of features for 4 h segments (resulting in reduced input dimensionality).

These advantages become particularly important for very sparse and very unbalanced seizure data.

For online prediction, this system requires additional modifications. They include:

• Periodic retraining, in order to address non-stationarity of iEEG data.
• Improving the quality of training data by removing training samples with noisy labels.
• A new post-processing scheme during prediction (test) stage.

Proposed system was evaluated using the long-term (169–365 days) iEEG recordings (i.e., American Epilepsy Society Seizure Prediction Challenge dataset) which were also used in previous studies [15,16,19,25,26,27,28,29,30].

This paper is organized as follows. Section 2 describes the definition of lead seizures, iEEG dataset and seizure clustering, and the proposed system for online seizure prediction. Experimental results demonstrating its prediction performance are presented in Section 3. Section 4 presents a discussion and summary.

## 2. Materials and Methods

### 2.1. Lead Seizures

Many earlier studies show prediction results for *all recorded seizures* [31,32]. Therefore, such studies report overly optimistic performance [27,28]. However, seizures are usually clustered (in time), and predicting the first one in a cluster (~lead seizure) is much harder.

**Figure 1:**
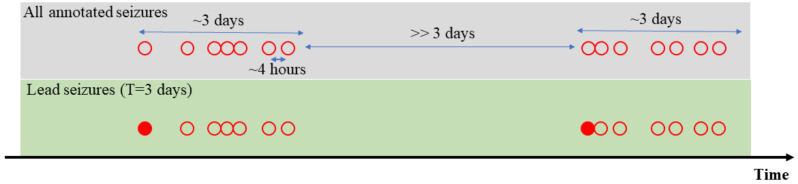
Lead seizures of seizure clusters. Fourteen seizures are annotated in the recording period (represented as 14 red circles); Using T = 3 days, two of them are defined as lead seizures (represented as filled red circles).

Many recent studies have acknowledged clinical significance of predicting lead seizures. Lead seizures are typically defined as seizures preceded by a certain (pre-defined) seizure-free period. The seizure-free period should be large enough to ensure lead seizures are the first seizure of seizure clusters (i.e., not following other seizures). Figure 1 shows an example of applying the large seizure-free period (T = 3 days) to specify lead seizures among annotated seizures. In this example, 14 annotated seizures form two seizure clusters. Applying the large T = 3 days can precisely specify the two first seizures (of the two seizure clusters) as lead seizures. However, there is still no consensus on the duration of this seizure-free period. Consequently, many studies apply a different length of the seizure-free period, even when using the same data set. For instance, for American Epilepsy Society Seizure Prediction Challenge dataset, several research teams apply different definitions of lead seizures, as summarized below:

• 4 h seizure-free period in Brinkmann et al. [26], Howbert et al. [27], Nejedly et al. [33], Varatharajah et al. [30].
• 80 min seizure-free period in Assi et al. [25], even though it was not explicitly stated in their paper.

Likewise, different definitions for lead seizures have been used for Melbourne-University AES-MathWorks-NIH Seizure Prediction Challenge dataset, e.g.,

• 8 h seizure free period was initially used in Cook et al. [34].
• Later, the same research team adopted a 4 h seizure-free period in Kuhlmann et al. [35].

Notably, none of these studies provide explanations for chosen specification of lead seizures. Clearly, using a shorter seizure-free period (for lead seizures) might not reflect the nature of seizure clusters, and might simplify the task of lead seizure prediction. That is, using shorter seizure-free period results in a larger number of lead seizures available for modeling, and thus better prediction performance. In addition, the seizures that happened earlier in the cluster could also be a hint for predicting the following seizures, which might contribute to inflated prediction performance.

In this study, lead seizures are specified in a data-driven fashion, based on cluster analysis [8]. That is, first, cluster analysis is applied to all seizure data recorded over long time period (294–475 days), and then lead seizures are identified as the first seizure in each cluster. This analysis is presented in Section 2.2. Remarkably, this analysis yields the *same 3 days seizure-free period* for all dogs, even though different dogs have different pattern of clusters.

### 2.2. Description of Available Intracranial Electroencephalogram (iEEG) Data and Analysis of Seizure Clustering

The proposed system is evaluated using canine iEEG data (American Epilepsy Society Seizure Prediction Challenge dataset) previously used in several seizure prediction studies [15,16,19,25,26,27,28,29,30]. For each dog, two bilateral electrode arrays containing eight channels were placed in the subdural space of the brain (one for each hemisphere) for sampling the iEEG signal. This 16-channel iEEG signal was continuously sampled at 400 Hz for a recording period of about 1 year. All the annotated seizures were automatically detected by a high-sensitivity seizure detection algorithm [36], and visually reviewed using both the iEEG signal and the continuous video to verify clinical seizure activity. The dataset is publicly available from Mayo Clinic (http://msel.mayo.edu/data.html, accessed on 7 Feb. 2017) and the iEEG portal (https://www.ieeg.org/, accessed on 7 February 2017). Canine data from epileptic dogs is used as a translational model for human seizure prediction, due to:

• Similarity of biological mechanisms of epilepsy in humans and dogs.
• Difficulty of obtaining long-term seizure recordings for humans (e.g., typical human iEEG data recording is 1–3 days long, corresponding to patients’ stay in a hospital).

This data set includes iEEG recordings from eight canines with naturally occurring epileptic seizures. Data sets containing too few seizures (<3) or having severe signal loss are not used in this study. The final data set (used for modeling) contains long-term iEEG recordings for four canines (labeled as L2, L7, M3, P2). Long-term recordings for each dog contain iEEG signals from 16 channels (electrodes). Table 1a shows the summary of iEEG data used for modeling, including the recording duration (days), the number of annotated seizures, and the number of gaps (i.e., interruptions in iEEG signal). Due to various technical reasons, such as device re-charging and emergency events, there are many discontinuities (called gaps) in available iEEG data. These gaps obviously affect the quality of recorded data and may degrade prediction performance; hence the duration of gaps is also recorded for each dog.

**Figure 2:**
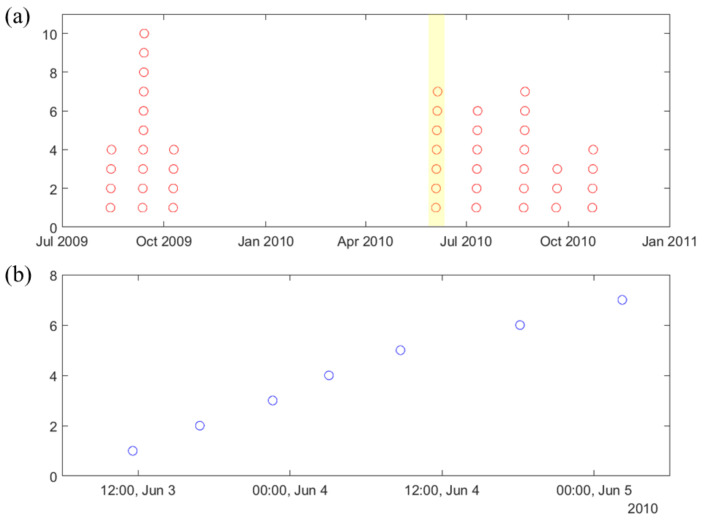
Clustering of seizures for canine data (L2): (**a**) distribution of all 45 seizures (in red circles) during the whole period (July 2009–November 2010), indicating strong clustering. (**b**) distribution of 7 seizures (in blue circles) within one cluster highlighted in (**a**), during time period 3–5 June 2010.

As stated earlier, seizures usually cluster (in time), and this paper is only concerned with the prediction of lead seizures, i.e., the first seizure (in a cluster). Proper specification of lead seizures should be performed based on clinical considerations and/or analysis of seizure distribution for each dog [8]. Such analysis of seizure clusters is presented next for dog L2 (see Figure 2). That is, Figure 2a shows the distribution of all annotated seizures during the whole recording period (July 2009–November 2010). It shows 45 annotated seizures (indicated as 45 red circles in Figure 2a) forming 8 seizure clusters that can be easily identified. The time interval between successive seizure clusters is about 1–2 months. Note that the long seizure-free period between third and fourth clusters (November 2009–June 2010) can be attributed to several large gaps during this period. Figure 2b shows the distribution of seizures within one cluster (highlighted in Figure 2a—showing seven annotated seizures with 5–12 h intervals between successive seizures in this cluster). Clearly, applying small value T = 4 h, as in previous seizure prediction studies [19,26,27,30,33], results in all seizures in Figure 2b regarded as lead seizures. However only the first seizure in this cluster should be considered ‘lead seizure’ based cluster analysis in Figure 2a. Other dogs (except dog M3) show similar clustering—as shown in Appendix A.

Statistical summary of seizure clusters (for all four dogs) is shown in Table 1b. This table indicates that seizures occur in clusters for all dogs, except for M3. Time duration of all clusters is in the range from 1 to 4 days; and average number of seizures per cluster is between 3 and 17. Based on observed time duration of clusters (for all dogs), we can use a 3-day seizure-free period for specification of lead seizures. This specification ensures that clusters are detected correctly (for all dogs). That is, all successive lead seizures for all dogs are separated by sufficiently long time (16–63 days). Also, note that the number of clusters (for different dogs) varies in the range 5–22, indicating subject-specific nature of seizure clustering.

For comparison, many earlier studies on seizure prediction used a 4 h seizure-free period to define lead seizures (without performing cluster analysis). Using a 4 h seizure-free period for dog L2 data will result in labeling *most annotated seizures* as lead seizures. In fact, all previous studies on online seizure prediction (using the same data for dog L2) define 85–100% of all seizures as ‘lead seizures’ [25,27,30,33]. In contrast, in this paper only 18% of all seizures for dog L2 are identified as lead seizures.

The 3-day seizure-free period for lead seizures will be used in this paper. It can be argued that this definition (of lead seizures) is more meaningful because it is based on natural clusters of seizures in real-life data [8]. However, using 3-day seizure-free period results in a smaller number of lead seizures, and hence in additional challenges for seizure prediction, including:

• Smaller number of labeled (preictal) training samples.
• Lower sensitivity, since lead seizures are harder to predict.

### 2.3. Online Modeling

*Online modeling*, also known as online seizure prediction, refers to a realistic modeling set-up when a classifier is trained using *only* past data, and then used for predicting future iEEG segments. Online modeling is inherently more difficult due to a small amount of available training data (compared to batch modeling). In addition, online modeling needs to address the non-stationarity of the iEEG signal. This non-stationarity results in two additional modifications:

• Training data should be selected from the *most recent* iEEG segments.
• A classifier needs to be *periodically retrained* (using most recent data).

**Figure 3:**
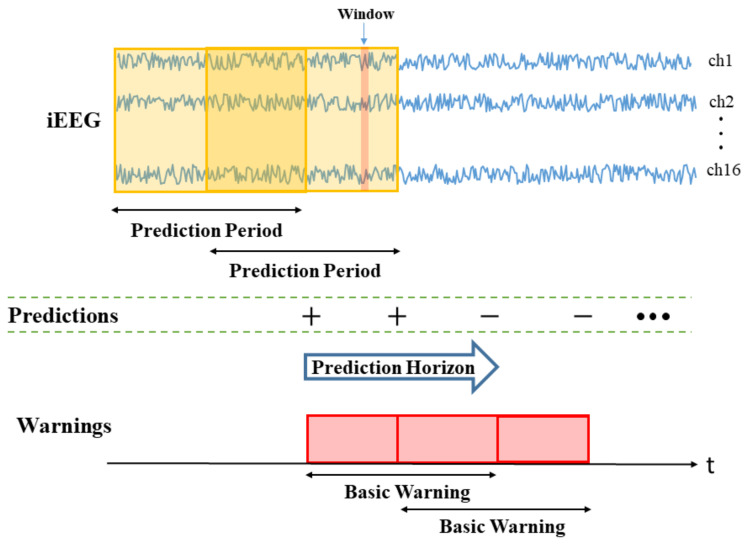
System design parameters for online prediction: window, prediction period and prediction horizon.

Furthermore, under online modeling, there are several important system design parameters during the prediction (test) period. These parameters refer to timing considerations for online prediction, as shown in Figure 3. These system parameters are discussed next:

• *Window size (W)* is the duration of iEEG signal used to classify preictal vs. interictal states. It reflects the change of brain state preceding seizures, and its typical range is 10–30 s (the value of W = 20 s is used in this paper). During the test stage, the trained SVM classifier makes predictions for all consecutive windows within prediction period (PP).
• *Prediction Period (PP)* is the length of the iEEG signal used for generating seizure prediction or warning during test stage. Its length obviously affects prediction performance; and different values of PPs were adopted in earlier studies (ranging from minutes to hours). Some studies do not clearly specify PP but effectively combine predictions from consecutive windows to generate single seizure prediction. The PP used in this study is 4 h, which equals the length of the iEEG segment used for training. The trained SVM classifier makes predictions for all windows within PP. Then predictions for all windows are combined via an adaptive post-processing scheme (described in Section 2.5.4) to make single prediction at the end of PP. These predictions are marked as positive (+) or negative (−) in Figure 3. A positive prediction indicates *seizure warning* for the duration of the next time period called prediction horizon (PH), whereas negative prediction indicates that there is no such warning. The system makes predictions every 2 h, so that each PP partially overlaps (50%) the previous one (see Figure 3).
• *Prediction Horizon (PH)* is the time period during which positive/negative prediction holds. That is, each positive prediction triggers a basic seizure warning for the fixed duration of PH. The length of PH is determined based on clinical considerations. Typical PH values are in the range 0.5–4 h. In this study, PH = 4 h is used.

Practical online prediction systems include *re-triggerable warning mechanism* [21], so that basic warnings can be combined. According to this re-triggering mechanism, consecutive basic warnings are combined (or extended) to form a longer warning (of variable duration). For example, Figure 3 shows two consecutive basic warnings that form a single warning of longer duration. A warning stops when a lead seizure is detected. Following the occurrence of a lead seizure, the system enters ‘high alert’ state for the duration of ‘minimum seizure-free period’. The operation of the seizure prediction system is resumed following this high-alert state.

### 2.4. Performance Metrics for Online Prediction

Typical performance indices for online prediction include sensitivity and specificity metrics evaluated during the *test period*.

Sensitivity is defined as the fraction of (lead) seizures accurately predicted during the test period:

(1) $$\mathrm{Sensitivity}=\frac{\mathrm{number}\;\mathrm{of}\;\mathrm{predicted}\;\mathrm{lead}\;\mathrm{seizures}}{\mathrm{total}\;\mathrm{number}\;\mathrm{of}\;\mathrm{lead}\;\mathrm{seizures}},$$

The specificity index is more difficult to evaluate due to small number of seizure events [31]. There are two commonly used performance indices related to specificity: time-in-warning (TIW) and false positive rate (FPR).

• TIW is defined as the duration of total warning time relative to total test period (i.e., the fraction of test period when the system generates warning).
• FPR is defined as the number of false warnings per day. In a re-triggerable warning system, this definition assumes that each warning may be of variable duration (as explained earlier).

These performance metrics are significantly affected by system design parameters [8]. For instance, the length of basic warning (equal to PH) directly affects TIW. That is, assuming that two systems make the same positive predictions (or basic warnings), the one with longer prediction horizon will have higher TIW. In other words, the performance of online seizure prediction system depends on *both* factors: (1) prediction accuracy of a binary classifier, and (2) system design parameters.

As evident from descriptions in Section 2.3, system design parameters (e.g., prediction period, prediction horizon, and minimum duration of seizure-free period) are inter-dependent, and their values cannot be specified arbitrarily.

### 2.5. Proposed System for Online Seizure Prediction

This section presents detailed description of the proposed system, and its empirical performance evaluation using canine iEEG data set.

First, it should be noted that seizure prediction is based on classification of short windows (e.g., 20 s long), even though the goal is to predict longer segments (say, 4 h long). Therefore, ‘predictions’ are made on two different time scales: for windows and for segments. This happens for two reasons:

• First, annotation of iEEG recordings is performed for segments, rather than individual windows. That is, original labeling of data (by medical experts) applies to segments.
• Second, classification (or model estimation) is performed for labeled windows, because (a) there are too few labeled segments, and (b) these segments are represented as high-dimensional feature vectors.

This mismatch between making predictions on two different time scales makes seizure prediction task very different from standard classification problem (in machine learning). Therefore, our system performs classification (for seizure prediction) using the so-called group learning method [16], where a classifier is trained using labeled windows data, and then during operation, predictions for group of windows are combined, in order to make prediction for the whole segment, i.e., for a group of consecutive windows.

**Figure 4:**
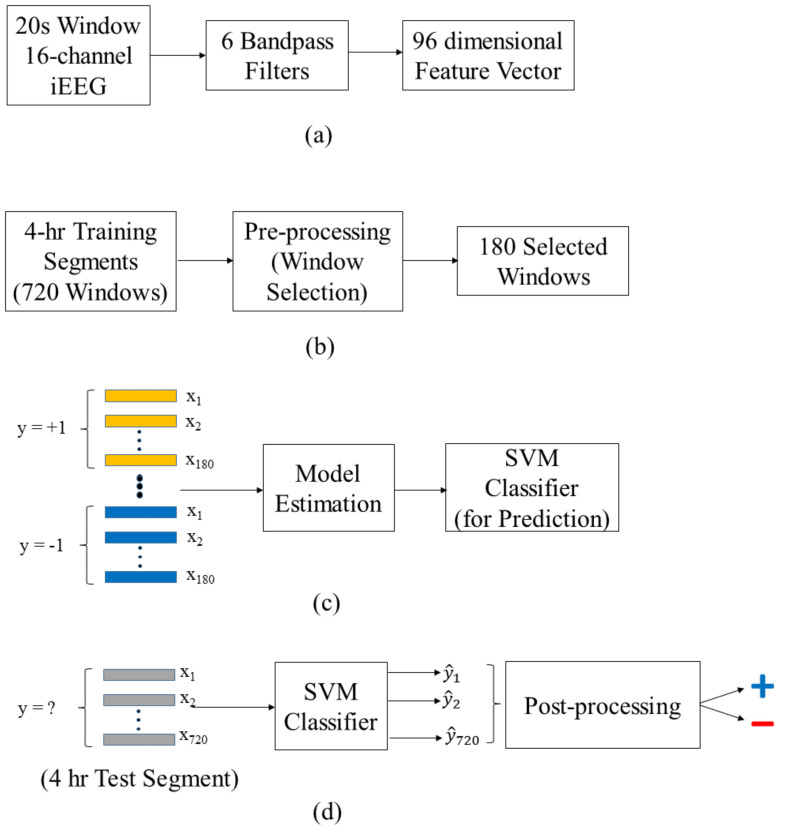
Overview of the proposed system: (**a**) feature encoding for 20 s windows; (**b**) preprocessing (~window selection); (**c**) model estimation (~SVM training); (**d**) test/prediction stage.

The main parts of the proposed system shown in Figure 4 include: (a) feature encoding for 20s windows, (b) selection of windows used for training, (c) training a classifier for windows, (d) testing or prediction stage. Here, the group learning approach is implemented using standard SVM classifier. A detailed description of the system is presented next.

Each 20 s iEEG window is represented as a 96-dimensional feature vector of the power in the six standard Berger frequency bands for 16 channels (see Figure 4a). Furthermore, each 4 h training segment is represented by its 720 windows, and pre-processing involves selection of the most informative 180 windows (out of 720 total—Figure 4b). Note that such window selection is *applied only* to labeled training segments (not test segments). During training stage, linear SVM classifier is estimated using these windows selected from all training segments, as shown in Figure 4c. Note that the classifier is trained for predicting 20 s windows. The classifier is periodically retrained (every 7 days), and then used for making predictions for the next 7-day test period. The retraining procedure is explained later in Section 2.5.2. During operation or the test stage (shown in Figure 4d), the system makes predictions for 4 h test segments. Predictions are based on classification of all 720 windows (comprising each 4 h segment). That is, SVM predictions for all 720 windows are combined to make single prediction for a 4 h test segment. This combining, aka post-processing method, is described later in Section 2.5.4.

#### 2.5.1. Data Preprocessing

First, we describe data preparation and feature selection used. Note that seizure data recorded within 50 days following electrode implantation was excluded from modeling due to severe non-stationarity immediately following surgery. The training data contain two classes of iEEG segments, preictal and interictal, which are used to train the SVM classifier. Preictal segments (or positive class) are labeled using a pre-specified ‘forecasting horizon’ (30 min in our system). That is, all the first 4.5–0.5 h period before seizures is labeled as preictal. This is so that a successful prediction could be made at least 30 min before the actual seizure. This is required for possible clinical intervention following seizure warning. All other (non-overlapping) 4-h segments are labeled as interictal (or negative class). Furthermore, all interictal segments within 3 days after a seizure are removed from the training data to ensure that interictal segments are sufficiently far away from a seizure.

As explained earlier in Figure 4a, each 20 s window is represented as a 96-dimensional feature vector. Specifically, each 20 s window is first passed through six bandpass filters corresponding to six standard Berger frequency bands (0.1–4, 4–8, 8–12, 12–30, 30–80, and 80–180 Hz). The output signals are then squared to obtain the estimated power in the six bands. All estimated powers from 16 channels form 16 × 6 = 96-dimensional feature vector. Each 4 h segment is represented as a group of 720 windows

The original labeling of iEEG recordings (by human experts) applies to 4 h segments, so that all 20 s windows (within this segment) are labeled accordingly. This labeling process of training windows presumes that the majority of windows within a segment have the same class label. This assumption may not be true, since human experts do not actually provide class labels for individual windows. Hence, poor labeling of 20 s windows (used for training) may result in poor classification (prediction) of test windows. To address this problem, we introduce new procedure for selecting most informative training windows, as detailed next.

Window selection is applied to a group of 720 windows comprising each 4 h labeled training segment, resulting in 180 selected windows (or 1-h training segment), as shown in Figure 4b. The window selection is performed using the real-valued outputs of a linear SVM classifier (trained using the original 4 h segment data, described next in Section 2.5.2). That is, for each 4-h training segment, we rank 720 SVM outputs (for all 720 windows), according to their distance from SVM decision boundary, and then select 180 windows most distant from the SVM decision boundary. This selection process ensures high confidence in classification labels of selected windows that are used later for model estimation (see Figure 4c).

#### 2.5.2. Training Stage

Model estimation (or training) is performed using labeled training segments, where each segment is represented as a group of windows. The same model estimation procedure is performed twice for two linear SVM models. The first SVM model for window selection is trained using the original 4 h segments (each of them contains 720 windows). The second SVM model for seizure prediction is trained using the selected 1 h segments (each of them contains 180 selected windows). Linear SVM is used for training a classifier, due to the small number of the preictal training sample (~preictal windows), and high dimensionality of the input space. LIBLINEAR package [37] is used for estimating SVM classifier.

Available seizure data are highly unbalanced and sparse, due to small number of seizures (over long-term recording time period). The classifier (in Figure 4c) is trained on heavily unbalanced data (i.e., interictal-to-preictal ratio is 8:1). The optimal value of the SVM regulation parameter C is selected from a range of values 0.0001 to 100,000. This optimal value corresponds to the minimum validation error estimated using M-fold cross-validation [19]. Here, M is the number of preictal segments in training data, which include M preictal and 16*M interictal segments. In each fold, validation data include one preictal segment and one interictal segment, and training data include the remaining preictal segments and interictal segments. This method for handling unbalanced training data is mathematically equivalent to training with unequal misclassification costs [22,38], or equivalently, to using subsampling or oversampling of training data from one of the classes [39,40]. There exist other approaches for unbalanced classification, that are tailored to specific learning algorithms [41,42,43,44].

**Figure 5:**
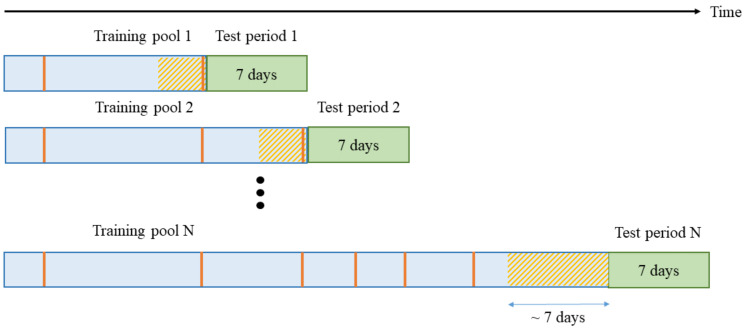
Retraining procedure: The classifier is retrained every week, and predictions are made for the next 7-day period (shown in green color). Vertical red lines indicate lead seizures. Training data contain most recent interictal segments (shown in shaded region). *Initial Training pool 1* contains 2 preictal segments and 16 interictal segments. *Training pool 2* is extended to include fresh interictal segments (for training). *Training pool N* contains 6 preictal segments, but only 5 recent ones are used for training.

In order to cope with the non-stationarity of iEEG signal, SVM classifier is periodically retrained (every 7 days). This retraining procedure is shown in Figure 5. The 7-day period was chosen because (a) it has sufficient number of interictal segments used for retraining, and (b) this period includes both day and night recordings, in order to capture variability of the iEEG signal during awake and sleep periods. The retraining procedure in Figure 5 implicitly defines the duration of the test period (7 days). That is, the system estimates SVM classifier using labeled training data (in the training pool), and then makes predictions for the next 7-day test period. During current round of retraining, the existence of seizures in the previous 7-day test period is known, so that the iEEG data in the previous 7-day test period can be labeled accordingly and included into next training pool, as shown in Figure 5. Initial training is performed using 2 preictal and 16 interictal segments (this initial training set is denoted as Training pool 1 in Figure 5). Note that only the most recent interictal segments are included in each training pool, in order to reflect non-stationarity of iEEG signal. These interictal training segments correspond to shaded region in Figure 5. Every 7 days, the training pool size increases to include most recent data for training. When the number of preictal segments (in the training pool) increases to more than five, only the most recent five are used for training. For example, consider Training pool N in Figure 5. It has six preictal segments, only 5 of which are used for training. For this training pool, the number of interictal training segments is 40, which covers approximately the whole recent 7-day period (i.e., forty 4 h segments ~6.7 days).

#### 2.5.3. Test Stage

System performance is evaluated over the whole recording period (after the initial training pool), called ‘total test period’, or just ‘test period’ in this paper. This test period corresponds to the union of all 7-day test periods (see Figure 5). Summary statistics for the test period (for different dogs) are shown in Table 2. The total test period is in the range of 169–364 days. However, the *net test period* is much shorter than the (nominal) test period because it excludes possible gaps. Note that the number of lead seizures during the test period is between 3 and 16, which is fewer than the number of lead seizures in Table 1b, because (a) two lead seizures are required for initial training, and (b) seizures that occur during the first 50 days following surgery are excluded. The classifier estimated during training stage is applied to iEEG data for making predictions during each 7-day test period.

As noted earlier in Section 2.3, both prediction period (PP) and prediction horizon (PH) are 4 h long. That is, predictions are made for every 4 h prediction period, where each PP corresponds to the duration of unlabeled test segment. Hence, during the test stage, 720 classifier predictions (for all 20 s test windows within a 4 h test segment) are combined together via post-processing to trigger a 4 h basic warning (~prediction for 4 h test segment)—see Figure 4d. The basic warning would last 4 h following a positive prediction to notify the patient a seizure is going to occur. The post-processing procedure is described next in Section 2.5.4. After a prediction for a basic warning is made, the prediction period slides 2 h forward, in order to make next prediction. Performance metrics (such as the false positive rate and time in warning) are calculated over ‘net test period’ (excluding gaps), rather than ‘nominal test period’. This results in very conservative estimates for TIW and FPR are reported later in Section 3.

#### 2.5.4. Adaptive Post-Processing for Group Learning

**Figure 6:**
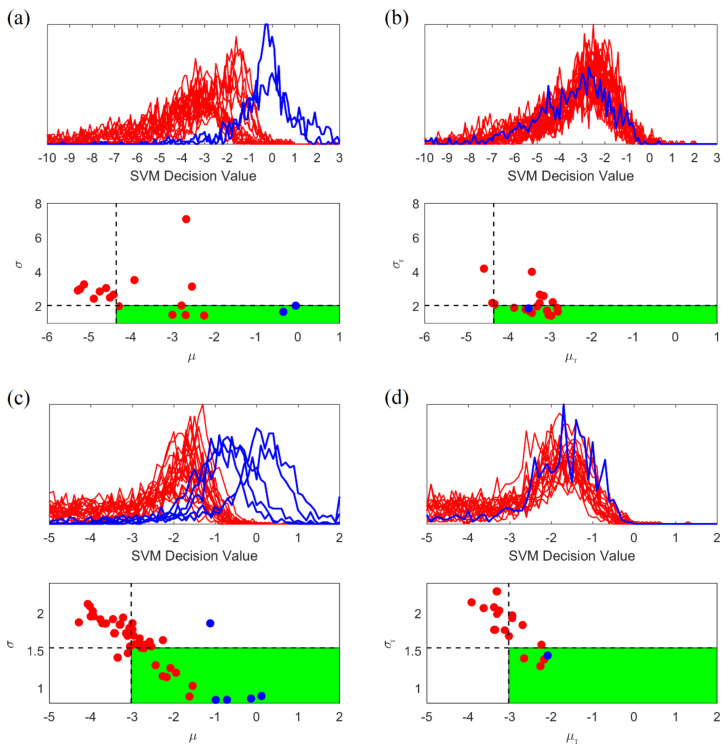
Histograms for 4 h iEEG segments and the corresponding bivariate decision space for discriminating between interictal (red lines/dots) and preictal (blue lines/dots) segments. Dashed lines denote thresholds, and green-shaded regions corresponds to preictal class segments. (**a**) Histograms for *training data* for Dog P2 (2 preictal and 16 interictal segments). (**b**) Histograms for *test data* for Dog P2 (1 preictal and 19 interictal segments). (**c**) Histograms for training data for Dog M3 (5 preictal and 40 interictal segments). (**d**) Histograms for test data for Dog M3 (1 preictal and 30 interictal segments).

Under the group learning approach, the system combines predictions for multiple 20 s windows into a single prediction for 4-h test segment. Hence, the main challenge during test stage is a method for combining SVM outputs/predictions for many consecutive windows into a single prediction for 4-h segment. Such a post-processing procedure should reflect global statistical characteristics of interictal and preictal training segments. Discrimination between interictal and preictal segments is a difficult task, due to highly overlapping class distributions. This can be better understood by looking at the distribution of distances between input feature vectors (for 20 s training windows) and the SVM decision boundary. These distances correspond to real-valued outputs of a trained SVM classifier. These real-valued SVM outputs are also known as ‘SVM decision values.’ The distribution of these distances can be visualized as a histogram formed by SVM outputs for all training windows (of a training segment). Each histogram corresponds to a single segment. Then histograms corresponding to all training segments, generated separately for preictal and interictal segments, can be used to visualize the difference between interictal and preictal class distribution (of SVM outputs). These histograms are shown in Figure 6a (top). Such histograms are also known as ‘histograms of projections’ [16,19,45]. Figure 6a (top) clearly illustrates two characteristics of iEEG data:

• High variability of histograms, especially for preictal segments.
• Large overlap between distributions (histograms) for preictal and interictal segments.

These two factors motivate the need to develop an *adaptive* decision-making mechanism (for seizure prediction) that incorporates *global* statistical characteristics of preictal and interictal data segments [16]. In the proposed system, adaptive post-processing is based on the mean and standard deviation of histograms for *interictal* training segments. That is, each histogram is represented by two numbers: mean and standard deviation of its real-valued SVM outputs, denoted as μ and σ. The distribution of the (μ, σ) values is shown in Figure 6a (bottom). Even though unknown distributions of distances (histograms) for interictal and preictal windows are heavily overlapping, their means and standard deviations are (relatively) stable, and they can be used for discriminating between the two distributions. However, distribution of SVM outputs can be *estimated only for interictal segments*, because there are too few preictal segments. Therefore, the discrimination rule for (unlabeled) test segments during post-processing is based on decision rule derived from the (μ, σ) values for (labeled) training interictal segments, as described next.

*Test segment* is classified as preictal (positive) if the mean and standard deviation of its histogram is *sufficiently different* from the distribution of the means/standard deviations (μ and σ) for *interictal training segments*. The notion of ‘sufficiently different’ is specified by *two adaptive quantile thresholds*, for the means and standard deviations of interictal distribution. This leads to the following post-processing decision rule for (unlabeled) *test segment T*:

1. **Generate the histogram of projections** corresponding to SVM outputs (predictions) for all consecutive windows within 4-h test segment. Then calculate its mean $\mu_{T}$ and standard deviation $\sigma_{T}$.
2. **Classify this unlabeled test segment** as preictal (positive) *if and only if*:
  • Its mean $\mu_{T}$ is larger than *50% quantile* of the μ values (for interictal training segments); and
  • Its standard deviation $\sigma_{T}$ is smaller than *30% quantile* of the σ values (for interictal training segments).

Note that adaptive thresholds (for μ and σ) are estimated only for interictal data (rather than preictal), because these thresholds can be reliably estimated only for the majority class (interictal data). For preictal (minority class) data, these thresholds show high variability, due to small sample size. Adaptive thresholds for quantile values (50% and 30%) have been optimally tuned for one dog (P2) training data, in order to achieve optimal trade-off between sensitivity (high) and time in warning (low) for that dog. These optimal thresholds yield the largest difference between sensitivity and TIW. Two adaptive thresholds reflect statistical properties of training data and they are applied to test data for making seizure prediction. *The same quantile values (50% and 30%)* optimized for P2, have been also used for all other dogs’ data.

Determination of these two thresholds for dog P2 is explained next. Figure 6a (top) shows histograms of projections for 2 preictal and 16 interictal training segments used for initial training for this dog. The distribution of (μ, σ) values for these histograms is shown in Figure 6a (bottom). In this decision space, one can tune adaptive thresholds for optimal separation between interictal and preictal training segments. Using such optimally tuned thresholds (*50% quantile* for μ and *30% quantile* for σ values of interictal segments), all preictal segments and the majority of interictal segments can be classified correctly, as shown in Figure 6a (bottom). The same thresholds (obtained from training data) are then used for making post-processing decisions for test segments. For dog P2, the histograms of projections for test segments (during a 7-day test period, 14–20 November 2012) are shown in Figure 6b (top), and the corresponding bivariate decision space for test segments is shown in Figure 6b (bottom). For the 7-day test period, a single preictal segment and about half of all interictal segments are predicted correctly—in spite of their heavily overlapping histograms. The heavily overlapping histograms are because these interictal segments are also very close to the seizure (~3 days), even though they were not labeled as preictal. However, interictal segments in other 7 days test periods (containing no seizures/far away from seizures) can be easily classified by the proposed system, proved by the low TIW and FPR shown next in Section 3.

Application of this post-processing procedure to a different dog (dog M3) is illustrated in Figure 6c,d. For this dog, Figure 6c shows good discrimination between interictal and preictal *training* segments, using the same quantile values (50% and 30%) for thresholds. The same adaptive threshold values also yield good separation between interictal and preictal *test* segments, as shown in Figure 6d (bottom). As evident from Figure 6c,d, the proposed adaptive post-processing decision rule yields robust discrimination between interictal and preictal segments, for both training and test segments. Notably, decision threshold values (for classifying test segments) are estimated using *only* training data. A similar conclusion regarding good performance of the proposed decision rule for post-processing holds for other dogs’ data sets.

## 3. Results

This section describes prediction performance results for the proposed system. All experiments follow the experimental set up described in Section 2. The test period (for each dog) is continuous iEEG recording (as shown in Table 2). The average test period (for all dogs) is 259 days and the average net test period is 140 days with an average of seven lead seizures (per dog) predicted—see Table 2. Prediction performance indices include: sensitivity (SS), false positive rate (FPR) per day, and time in warning (TIW).

In addition, Section 3.2 describes performance reported in other seizure prediction studies using the same data set [25,26,27,30,33]. However, direct comparisons may be misleading, because they use different specification for system design parameters. In particular, using shorter seizure-free period for specification of lead seizures usually results in improved performance indices, because non-lead seizures are much easier to predict. This phenomenon is discussed in Section 3.2.

### 3.1. Prediction Performance of Online Prediction System

Prediction performance of the proposed system is summarized in Table 3a, showing sensitivity, false positive rate (FPR) per day, and time in warning (TIW) for each dog. These results show good overall performance, i.e., high sensitivity (0.84 on average), low FPR per day (0.71 on average), and low TIW (0.27 on average). Furthermore, careful examination of results for dog L7 indicates that the only mis-predicted seizure occurred following the 2-month gap in iEEG recording. Hence, this particular seizure was very difficult to predict, because the classifier should be retrained weekly, using most recent interictal data (see Figure 5).

Note that according to experimental procedure (shown in Figure 5), during initial training, the number of preictal segments is smaller than five. That is, initial Training Pool 1 contains two preictal segments, Training Pool 2 has three preictal segments, etc. For these initial training pools, prediction accuracy is typically lower than for later training pools (containing five preictal segments). For example, prediction model for Dog L2 estimated using initial training pool (having two preictal segments) shows FPR of 1.01; however, when the number of preictal training segments is increased to five, FPR is reduced to 0.75. Similar improvement in FPR (with increasing number of preictal training segments) can be observed in other dogs.

To investigate performance improvement due to window selection (in the training set), we compare prediction performance using training data with and without window selection. We remove the window selection procedure from the online seizure prediction system and repeat the same experiment. The results in Table 3b (~prediction without window selection) show the same sensitivity for all dogs; however, both FPR and TIW are higher than for the system with window selection (see Table 3a). Therefore, the proposed method for selecting informative training windows can indeed reduce the number of false positives and the warning time without decreasing sensitivity. Window selection also helps us to achieve more robust predictions, which can be seen from smaller variability of FPR and TIW (see Table 3a).

Performance results in Table 3a were obtained using most recent interictal data in the training pool (see Figure 5). Using the most recent interictal data for training reflects the assumption of non-stationarity of the iEEG signal. In order to validate this assumption, we performed another set of experiments, in which interictal training segments were selected randomly from the whole training pool (see Figure 5). Under this scenario, the training pool contains ‘old’ interictal data. Each experiment is repeated five times and average prediction performance for each dog is presented in Table 4. Comparison of Table 4’s results with Table 3a indicates that: the false positive rate (FPR) and time in warning (TIW) remain low, but the sensitivity has decreased very significantly, suggesting degradation in performance (vs. results in Table 3a). Furthermore, all performance indices (Sensitivity, FPR, and TIW) vary greatly (as shown by their high standard deviation). This can be explained by non-stationarity of iEEG data. That is, for a long-term training pool (~several months), randomly selected interictal segments will likely have very different statistical characteristics. These experiments suggest that using the most recent (fresh) interictal data for training yields better performance.

As explained earlier in Section 2.3, our online prediction systems include *re-triggerable warning mechanism* [21], such that consecutive basic warnings are combined to form a longer warning (of variable duration). Therefore, another important statistic is a duration of this longer combined warning. In the proposed system, the average time between the first warning until seizure occurrence is given below (for each dog):

For dog L2 ~6.7 h, dog L7 ~11.5 h, dog M3 ~13.8 h, dog P2 ~14 h. These statistics may be useful because they effectively show the duration of the warning period (prior to leading seizure). This duration is sufficiently long for possible clinical interference/treatment, following the initial warning.

### 3.2. Other Online Seizure Prediction Studies

This section describes prediction performance results achieved in other studies using the same iEEG data set [25,26,27,30,33]. However, these studies use very different experimental set-ups, data selection, and specifications of system parameters. Therefore, meaningful direct comparisons cannot be made, even when using the same iEEG recordings data. These differences are summarized in Table 5.

With regard to specification of iEEG data used for modeling:

• Our study includes four canines with the *same number* of iEEG channels (16 channels). One canine (with severe signal loss in an entire channel) is not used for modeling.
• Brinkmann et al. [26] and Varatharajah et al. [30] use all five canines’ data for modeling.
• Nejedly et al. [33] use four canines’ data, including one dog with a missing channel. On the other hand, dog P2 with the smallest number of seizures is excluded from their study. This results in the largest number of seizures (available for modeling), and improves overall prediction performance.
• Howbert et al. [27] and Assi et al. [25] include only three canines for modeling. Furthermore, they use only a portion of available iEEG recording for modeling.

With regard to the specification of lead seizures:

• Our study uses 3-day seizure free period to define lead seizures. This is based on the previous study [8] and the statistical analysis of seizure clusters presented in Section 2.2.
• Brinkmann et al. [26], Varatharajah et al. [30], and Nejedly et al. [33] use a 4 h seizure free period to define lead seizures. This results in a much larger number of lead seizures, and simplifies the task of predictive modeling.
• Assi et al. [25] apply only an 80 min seizure free period to define lead seizures. So, they have a large number of lead seizures, even though they use just a portion of iEEG recordings.
• Howbert et al. [27] apply both 4 h and 80 min seizure free periods to define lead seizures for comparing the effect of using different of lead seizures on prediction performance.
• As a result of these differences, each study uses a different average number of lead seizures (as shown in Table 5).

In view of these differences (summarized in Table 5), it is not possible to compare directly prediction performance reported in these studies. Nevertheless, we briefly discuss how prediction performance is affected by different selection of system design parameters. For example, using smaller T-values results in better prediction performance. This is supported by results shown in [27] who compared prediction performance using different values for T (~4 h and 80 min). As shown in Table 5, their system achieves higher sensitivity for T = 80 min. Another study [25] uses the same data set. However, they do not consider the gaps in raw iEEG recordings, which results in an underestimation of the value of TIW. For instance, for dog L7 recording, the portion of gaps is about 2/3 of the total recording period (see Table 2). Therefore, without adjusting for gaps, the reported value of TIW is only 1/3 of the ‘true’ TIW (excluding gaps).

**Figure 7:**
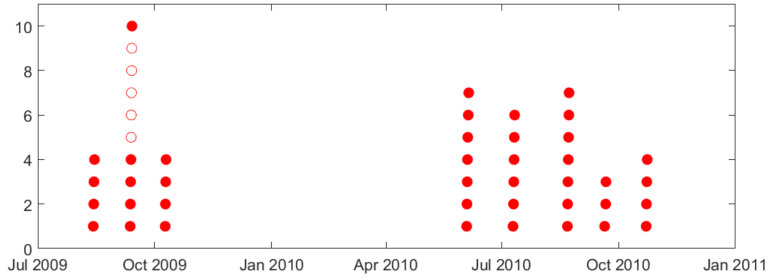
Lead seizures (defined by T = 4 h) for dog L2 during observation period (July 2009–November 2010). Each circle represents an annotated seizure; shaded circles indicate 40 lead seizures (out of 45 total).

**Figure 8:**
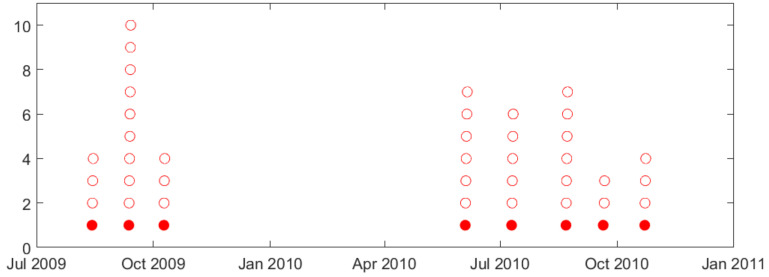
Lead seizures (defined by T = 3 day) for dog L2 during observation period (July 2009–November 2010). Each circle represents an annotated seizure; shaded circles indicate 8 lead seizures (out of 45 total).

In earlier studies, the duration of seizure-free period (used to specify lead seizures) is typically small (~4 h or 80 min), and does not reflect clustering of seizures in iEEG recordings. Analysis of natural clusters in available data suggests the proper duration T = 3 days for this canine dataset [8]. Clustering of seizures and its effect on proper selection of seizure-free period is illustrated next using dog L2 data. Both Figure 7 and Figure 8 show distribution of all annotated seizures (in time) over a long-term observation period; and one can clearly identify seizure clusters (in time). For this data set, using a 4 h seizure-free period, most annotated seizures will be regarded as lead seizures, as shown in Figure 7. On the other hand, using a 3-day seizure-free period clearly identifies a lead seizure as the first seizure in a cluster (see Figure 8). It is also worth noting that the 3-day seizure period applies to *all dogs*, e.g., seizure clusters are separated by (at least) 3 days for all data sets. This similarity of clustering in different dogs reflects certain biological/clinical mechanisms of epilepsy. That is, for most epileptic patients, seizures occur in clusters, i.e., in close succession (in time).

Arguably, using small T-values (~80 min and 4 h) in previous studies [25,26,27,30,33] significantly simplifies the task of seizure prediction. This is because all seizures within a cluster can be reliably predicted, after the first leading seizure is observed. Following this argument, Chen and Cherkassky [8] suggested a simple fixed ‘prediction rule’ that will always mis-predict the first/lead seizure in a cluster, but then successfully predict *all other seizures in a cluster*. For this canine dataset, such a simple rule will achieve 0.75–0.80 sensitivity (for lead seizures defined using small T = 4 h), because there are 4–6 seizures in each cluster (e.g., see Figure 7 and Table 1b). In fact, such a sensitivity is *similar or better* than reported in [25,30,33]. Moreover, this fixed rule will correctly predict most interictal segments between clusters and thus achieves *similar or lower* TIW than reported in [25,30,33].

Selecting an optimal threshold for triggering a warning controls the trade-off between sensitivity and specificity, or between sensitivity and TIW (or FPR). Performance results in our paper were obtained under a more conservative (and more realistic) experimental set-up compared to previous studies [26,27,30]. In all these studies, TIW value is pre-set at 0.30 and then the threshold is fine-tuned to achieve the highest possible sensitivity (~0.70). Arguably, such approach yields inflated performance results, because (a) future (test) data are used to fine-tune the threshold (which is a form of overfitting), and (b) optimal threshold values are patient-specific.

Our paper follows a different approach:

First, a threshold is estimated using one dog’s data, in order to achieve the largest difference between sensitivity and TIW (for this dog). The same threshold is then used for all other dogs. This approach (using fixed threshold) avoids overfitting, and should result in more conservative performance results (than previous studies using the same data). Nonetheless, the proposed system still can achieve sensitivity ~0.84, TIW ~0.27, and FPR ~0.78, which are very competitive (in terms of the performance indices) with previous studies.

Typical values of TIW (~0.20–0.30) presented in seizure prediction studies do not appear to be very useful in practice. Therefore, future seizure prediction systems should include other informative inputs, in addition to EEG signals. Sometimes, the trade-off between sensitivity and TIW can be adjusted to achieve lower TIW (at the cost of sensitivity). That is, patients or clinicians can vary the threshold for preferred performance indices. For example, during sleep/night time, there is no risk of a patient falling down, so sensitivity can be lower than during day time.

## 4. Discussion

We described a system for online prediction of lead seizures from iEEG signals. To the best of our knowledge, the canine dataset used in our study is the only publicly available long-term set of iEEG recordings (>one year) for seizure prediction. Hence, our system is tested only using this canine dataset. The system was tested using long-term recordings (169–365 days), and it achieves competitive prediction performance (relative to other studies). Remarkably, the proposed system can use only two lead seizures for initial training. Such learning using just a few labeled examples is difficult for most existing methods (e.g., Deep Neural Networks). The non-stationarity of the iEEG signal and its variability due to subject-specific modeling makes the task of seizure prediction very challenging. In order to address these problems, the system applies periodic retraining (every 7 days) using most recent data, a new method for selecting informative windows for training, and incorporates new adaptive post-processing during testing (prediction) stage. These contributions are summarized next. In addition, we discuss several methodological issues important for all seizure prediction studies.

### 4.1. Quality of iEEG Data

For the long-term recordings (about 1 year), it is difficult to maintain consistent quality of available iEEG data. Actual recordings include a considerable number of gaps (i.e., interruptions), for various reasons such as maintenance of electrical devices, moving dogs from one location to another, etc. It is impossible to assess the effect of these gaps on prediction performance. Our empirical results clearly show degradation in prediction performance when a seizure occurs immediately following a long gap, such as a single mis-predicted seizure for dog L7 (following two-month gap).

### 4.2. Non-Stationarity and Retraining

Non-stationarity of an EEG signal is potentially a major problem for online seizure prediction via data-analytic modeling [46]. In our system, this problem is addressed by periodic retraining (every 7 days)—effectively assuming *slow changes* in statistical characteristics of iEEG signal.

Many earlier studies did not use retraining because:

(a) some studies used short-term recording period. For short-term recording period (~several days), the problem of non-stationarity of EEG signal is not as severe as for long-term recording (~several months).
(b) these studies used batch modeling, where the problem of non-stationarity is alleviated because future data may be used for training.

However, for realistic online prediction using long-term recordings, non-stationarity becomes critical. Cook et al. [34] mentioned a retraining strategy (once every 4 months) for modeling long-term recordings of human iEEG data. Unfortunately, the effect of retraining is not reported since they only focus on the prediction performance during the first 4-month period.

In our paper, empirical results demonstrate that periodic retraining using most recent data in the training pool helps to improve prediction performance. Clearly, an ‘optimal’ length of a retraining period can be affected by various factors, such as the duration of long-term recordings, sensor technology (for iEEG recordings), the length of prediction horizon, and specification of lead seizures.

### 4.3. Improving the Quality of Training Data

All seizure prediction studies [15,16,19,22,26,27,30,33] assign binary labels (interictal/preictal) to iEEG segments (~0.5–4 h), based on seizure events annotated by human experts. However, human experts cannot label individual shorter windows that are used for classification. Therefore, all windows (~10–60 s long) within a segment are assigned the same label (preictal or interictal). As mentioned in Section 2.5.1, this procedure results in many mis-labeled windows and thus very noisy training data. That is, class distributions corresponding to interictal and preictal data are highly overlapping.

In the proposed system, this inherent deficiency of the labeling process has been (partially) addressed by selecting/using only informative windows for training. The proposed method effectively checks for consistency of labeling for all 20 s windows within a given training segment, and then only windows with consistent labels are used for training. This technique improves prediction performance, e.g., it achieves lower TIW for all canines while keeping sensitivity high.

### 4.4. Adaptive Post-Processing

In the proposed system, a new post-processing method is used to combine predictions of all 20 s windows within a prediction period. The combining method considers global statistical characteristics of all predictions (~SVM outputs) for individual windows.

This is achieved by representing SVM outputs for all windows (of a test segment) as a ‘histogram of projections.’ Then, classification of test segments amounts to discriminating between histograms for interictal vs. preictal segments. This is a challenging problem, because distributions of histograms for the two classes are heavily overlapping. However, as shown in this paper, robust classification of test segments is still possible, using global statistical indices of histograms for training segments.

Our paper provides specific post-processing rules for discriminating between interictal and preictal test segments using two adaptive thresholds. The same rules (for thresholds) were used for all dogs. Arguably, it may be possible to introduce subject-specific thresholds as well. This can further improve prediction performance.

### 4.5. Methodological Issues

There are several methodological aspects important for all online seizure prediction systems:

*Sound experimental set-up* for predictive modeling. For example, experimental set-up should not use test data for model estimation (including tuning of decision thresholds).

*Specification of lead seizures*. The notion of lead seizures should be based on clinical considerations, and also reflect natural clustering of seizures (in time), that can be estimated from patients’ past history [8]. Using this method, we observed strong and consistent clustering of annotated seizures, and then defined lead seizures as the first seizure in each cluster.

*Specification of system design parameters*. These parameter values obviously affect performance indices for seizure prediction. However, such parameters have to be specified prior to modeling, rather than tuned to achieve optimal system performance. Therefore, objective performance comparison (of different systems) on the same iEEG data, can be made only when using the same values of system parameters, that are pre-specified based on clinical considerations. Design parameters for online prediction include: prediction period PP, prediction horizon PH, and forecasting horizon FH. Forecasting horizon (FH) is determined by clinical considerations, with typical values in the range of 5–30 min. Selection of PH and PP parameters affects prediction performance. A longer PH results in higher sensitivity, but also increased time for warning. A shorter prediction period (PP) results in a larger false positive error rate, because predictions using shorter test segments are more prone to errors [8]. In our paper, the values of PP and PH are both set to 4 h, which is consistent with the length of interictal/preictal segments used during training.

*Other predictive inputs*. Most systems for seizure prediction use only iEEG signals for prediction. In practice, there are many additional informative inputs that can be used to improve prediction. For example, one can use sleep/awake indicators and heart rates as additional inputs. Combining these additional inputs with iEEG signals is expected to improve prediction performance.

## Figures and Tables

**Table 1:** (**a**) Summary of iEEG recordings. (**b**) Summary of seizure clusters.

(a)				
Dog ID	Recording Duration (Days)	Number of Annotated Seizures	Number of Gaps (>7 Days)	Number of Gaps (>1 h)
L2	475	45	4	87
L7	451	105	4	26
M3	394	29	4	155
P2	294	22	7	93
**(b)**				
**Dog ID**	**Cluster Time** **Duration** **(Days)**	**Number of** **Seizures in Each Cluster (Ave.)**	**Number of Lead Seizures**	**Time between Lead Seizures (Ave. Days)**
L2	1–2	5.6	8	62.2
L7	1–2	17.2	8	62.9
M3	1–4	2.8	22	16.6
P2	0–1	4.4	5	33.6

**Table 2:** Summary statistics for test period.

Dog ID	Test Period (Days)	Net Test Period (Days)	Number of Lead Seizures
L2	169	149	4
L7	364	123	5
M3	320	245	16
P2	183	42	3
Average	259 ± 98	140 ± 84	7.0 ± 6.1

**Table 3:** Performance of seizure prediction system with and without window selection. (**a**) Prediction performance of online seizure prediction system with window selection. (**b**) Prediction performance of online seizure prediction system without window selection.

(a)			
Dog ID	Sensitivity	FPR/Day	TIW
L2	0.75 (3/4)	0.85	0.27
L7	0.80 (4/5)	0.54	0.25
M3	0.81 (13/16)	0.70	0.29
P2	1.00 (3/3)	0.73	0.27
Average	0.84 ± 0.11	0.71 ± 0.13	0.27 ± 0.02
**(b)**			
**Dog ID**	**Sensitivity**	**FPR/Day**	**TIW**
L2	0.75 (3/4)	0.96	0.40
L7	0.80 (4/5)	0.57	0.29
M3	0.81 (13/16)	0.88	0.37
P2	1.00 (3/3)	0.71	0.30
Average	0.84 ± 0.11	0.78 ± 0.17	0.34 ± 0.05

**Table 4:** Average prediction performance using interictal data randomly selected from the whole training pool (five repeats).

Dog ID	Sensitivity	FPR/Day	TIW
L2	0.35 ± 0.49	0.61 ± 0.27	0.26 ± 0.21
L7	0.64 ± 0.30	0.50 ± 0.11	0.35 ± 0.16
M3	0.46 ± 0.14	0.63 ± 0.18	0.26 ± 0.14
P2	0.67 ± 0.00	0.59 ± 0.19	0.19 ± 0.08
Average	0.53 ± 0.15	0.58 ± 0.06	0.26 ± 0.07

**Table 5:** Experimental settings used in different studies. Average performance indices are shown.

Study	Number of Canines	Average Number of Lead Seizures	Sensitivity	TIW	FPR (Per Day)
**Parameter T for lead seizures = 3 days**					
This paper	4	10.8	0.84	0.27	0.78
**Parameter T for lead seizures = 4 h**					
Howbert et al. [27]	3	17.7	0.60	0.30	2.06
Brinkmann et al. [26]	5	35.8	0.69	0.30	1.10
Varatharajah et al. [30]	5	35.8	~0.70 †	0.25	—
Nejedly et al. [33]	4	42.8	0.79	0.18	—
**Parameter T for lead seizures = 80 min**					
Howbert et al. [27]	3	41.7	0.79	0.30	2.06
Assi et al. [25]	3	41.7	0.85	0.10	—

† Exact number is not provided, the value of sensitivity ~0.70 has been inferred from the figures presented in [30].

**Figure A1:**
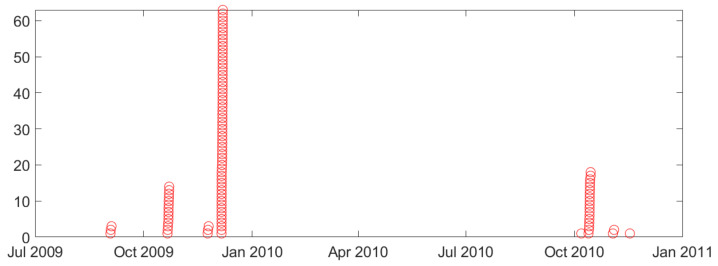
Clustering of seizures for canine data (L7): *Explanation:* among all 105 seizures (shown in red circles), 103 seizures occur in clusters, 2 seizures are isolated. Using T = 3 days, 8 clusters (~lead seizures) can be detected. Cluster duration is between 1 to 2 days, duration between two lead seizures is between 6 and 304 days.

**Figure A2:**
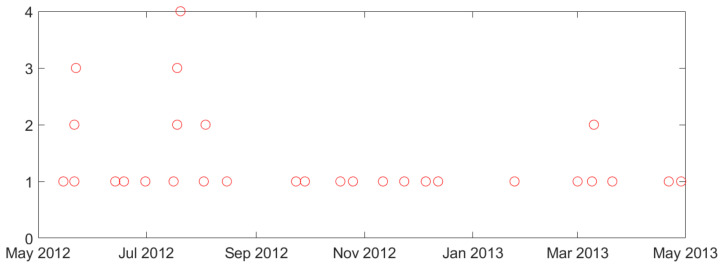
Clustering of seizures for canine data (M3): *Explanation:* among all 29 seizures (shown in red circles), 11 seizures occur in clusters, 18 seizures are isolated. Using T = 3 days, 22 clusters (~lead seizures) can be detected. Cluster duration is between 1 to 4 days, duration between two lead seizures is between 5 and 43 days.

**Figure A3:**
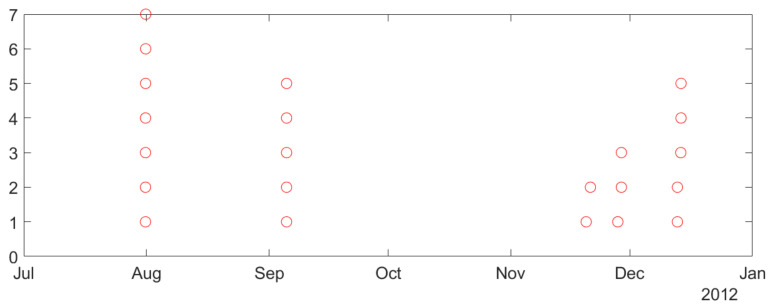
Clustering of seizures for canine data (P2): *Explanation:* among all 22 seizures (shown in red circles), All 22 seizures occur in clusters. Using T = 3 days, 5 clusters (~lead seizures) can be detected. Cluster duration is between 0 to 1 days, duration between two lead seizures is between 8 and 75 days.

## Data Availability

The data presented in this study are available on https://www.ieeg.org/ (accessed on 7 February 2017) or request from the corresponding author.
